# Biological Impact of Silicon Nitride for Orthopaedic Applications: Role of Particle Size, Surface Composition and Donor Variation

**DOI:** 10.1038/s41598-018-27494-y

**Published:** 2018-06-14

**Authors:** Saurabh Lal, Emily A. Caseley, Richard M. Hall, Joanne L. Tipper

**Affiliations:** 1 School of Biomedical Sciences, University of Leeds, LS2 9JT Leeds, UK; 2 School of Mechanical Engineering, University of Leeds, LS2 9JT Leeds, UK

## Abstract

The adverse biological impact of orthopaedic wear debris currently limits the long-term safety of human joint replacement devices. We investigated the role of particle size, surface composition and donor variation in influencing the biological impact of silicon nitride as a bioceramic for orthopaedic applications. Silicon nitride particles were compared to the other commonly used orthopaedic biomaterials (e.g. cobalt-chromium and Ti-6Al-4V alloys). A novel biological evaluation platform was developed to simultaneously evaluate cytotoxicity, inflammatory cytokine release, oxidative stress, and genotoxicity potential of particles using peripheral blood mononuclear cells (PBMNCs) from individual human donors. Irrespective of the particle size, silicon nitride did not cause any adverse responses whereas cobalt-chromium wear particles caused donor-dependent cytotoxicity, TNF-α cytokine release, oxidative stress, and DNA damage in PBMNCs after 24 h. Despite being similar in size and morphology, silicon dioxide nanoparticles caused the release of significantly higher levels of TNF-α compared to silicon nitride nanoparticles, suggesting that surface composition influences the inflammatory response in PBMNCs. Ti-6Al-4V wear particles also released significantly elevated levels of TNF-α cytokine in one of the donors. This study demonstrated that silicon nitride is an attractive orthopaedic biomaterial due to its minimal biological impact on human PBMNCs.

## Introduction

Silicon nitride is an industrial ceramic used for high-performance applications such as heat exchangers, gas turbines, and automotive engines due to its excellent temperature resistance, superior mechanical strength, relatively high toughness, and abrasion resistance^1^. In addition to these mechanical properties, bulk silicon nitride (Si_3_N_4_) has been shown to be a biocompatible, osteoconductive^2–4^, and an anti-infective material^5^. This has allowed the clinical use of Si_3_N_4_ for spinal fusion devices. Currently, monolithic Si_3_N_4_ and ceramic-like silicon nitride coatings are actively being investigated as potential bearing materials for orthopaedic reconstruction devices such as total hip replacements^6,7^.

It is increasingly evident from recent studies that Si_3_N_4_ surface chemistry plays an important role in its biological identity and interaction with cells^4,8^. Moreover, it has been reported by a number of investigators that in an aqueous environment a hydrated layer of silicon dioxide (SiO_2_) is formed on the Si_3_N_4_ bearing surfaces^9–11^. Therefore, in addition to the generation of Si_3_N_4_ particles, tribochemical wear of Si_3_N_4_ articulating surfaces could release particles with a surface composition similar to SiO_2_. Currently, tribochemical wear has been observed in hard-on-hard Si_3_N_4_ bearings and ceramic-like coatings^6,12,13^. Tribochemical dissolution of Si_3_N_4_ also offers a unique potential advantage, in that the wear particles that are released are slowly dissolved in biological fluids, reducing the overall particle load over the long-term use of a device^14,15^.

Wear is an important factor that affects the long-term clinical performance of orthopaedic reconstruction devices. As evident from metal-on-UHMWPE bearings^16^, excessive release of particulates could lead to a range of biological responses, eventually causing aseptic loosening of a device. Moreover, the volumetric dose and size of particles are known to influence osteolysis^17^. In metal-on-metal bearings, excessive release of nanoscale cobalt-chromium (CoCr) wear particles and ions from metal-on-metal bearings leads to a range of conditions termed as Adverse Reactions to Metal Debris (ARMD)^18^. Long-term joint registry data also indicates that aseptic loosening, implant wear, and adverse reactions to particulate debris are the key reasons for hip and knee revision surgeries^19^. Therefore, any new orthopaedic biomaterial such as Si_3_N_4_ requires thorough testing to determine if its particulates potentially induce adverse responses in humans.

Pre-clinical biological assessment of orthopaedic wear particles typically consists of *in vitro* cytotoxicity testing and evaluation of inflammatory cytokine release^20–22^. Although established cell lines such as L929 fibroblasts are commonly used for *in vitro* biocompatibility testing of particles, primary human Peripheral Blood Mononuclear Cells (PBMNCs) challenged with clinically relevant doses of particles offer a physiologically relevant means of testing the biocompatibility of wear particles released from joint replacements^21,23,24^. Moreover, evaluation of key indicators such as cell viability, inflammatory cytokine release, oxidative stress, and DNA damage can provide a thorough assessment of the biological impact of a material that goes beyond biocompatibility testing.

*In vitro* evaluation of Si_3_N_4_ has been carried out in the past using human and murine cell lines by direct-contact cytotoxicity^2,3,25^, osseointegration^5^, and inflammatory cytokine release^25^. Most of these studies investigated the biological interactions of Si_3_N_4_ as a porous^26^ or dense bulk material^3,27^. There is currently very limited information available about the biocompatibility of Si_3_N_4_ as particulate debris from orthopaedic implants^25^. In addition, previous studies did not use human PBMNCs challenged with clinically relevant doses of particles, nor did they investigate the influence of particle size or surface composition on the biological impact of Si_3_N_4_.

The aim of this study was to perform comprehensive evaluation of the biological impact of Si_3_N_4_ particles using a novel biological evaluation platform capable of using PBMNCs from individual donors for testing cytotoxicity, inflammatory cytokine release, oxidative stress, and the genotoxicity potential of particles simultaneously at clinically relevant low (0.5 μm^3^ per cell) and high (50 μm^3^ per cell) doses. Using this approach, the biological impact of Si_3_N_4_ was tested with a set of objectives. Firstly, the effect of particle size was evaluated by comparing the biological responses to nanoscale and micron-scale Si_3_N_4_ particles. Secondly, the influence of surface composition was investigated by comparing Si_3_N_4_ and SiO_2_ nanoparticles with comparable size and morphology. Thirdly, the biological responses to cobalt-chromium and titanium were investigated as they are likely to be the substrate material of choice, where silicon nitride is used as a coating material for orthopaedic implants^28,29^. Finally, donor variation was investigated by using PBMNCs from three healthy human donors.

## Results

### Characterisation of the particles

**Figure 1:**
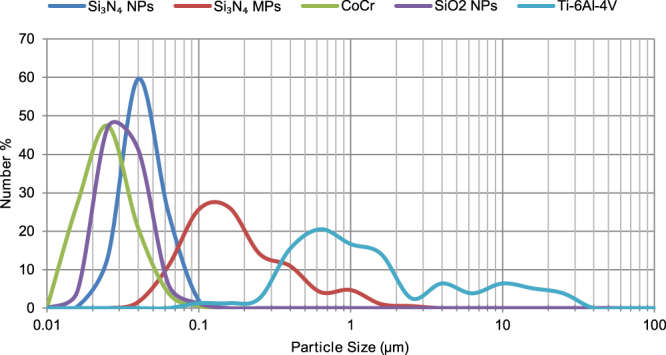
Particle size distributions of silicon nitride nanoparticles (Si_3_N_4_ NPs), silicon nitride micron-scale particles (Si_3_N_4_ MPs), cobalt chromium wear particles (CoCr), silicon dioxide nanoparticles (SiO_2_ NPs) and Ti-6Al-4V wear particles.

**Figure 2:**
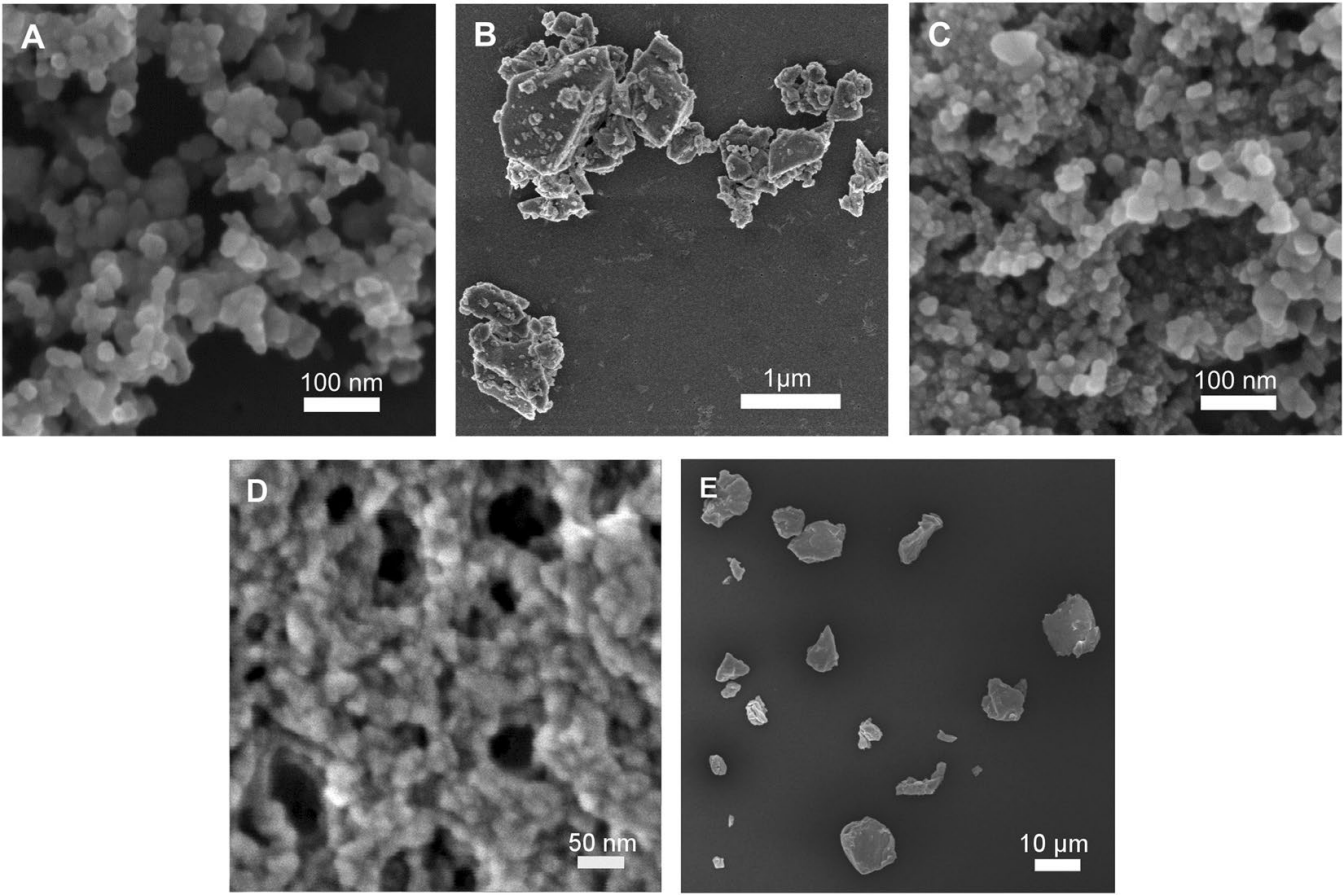
Representative scanning electron micrographs of nanoscale and micron-scale particles. (**A**) Si_3_N_4_ nanoparticles. (**B**) Si_3_N_4_ micron-scale particles. (**C**) SiO_2_ nanoparticles. (**D**) CoCr wear particles. (**E**) Ti-6Al-4V wear particles.

The majority of the nanoscale particles from the three materials (Si_3_N_4,_ SiO_2_ and CoCr) were between 10 and 100 nm in size (Fig. 1) and had a spherical or spheroidal-smooth morphology (Fig. 2). Moreover, Si_3_N_4_ nanoparticles had the highest mean particle size and mean roundness among all three types of nanoparticles (Table 1).

**Table 1:** Particle size, aspect ratio and roundness data for silicon nitride nanoparticles (Si_3_N_4_ NPs), silicon nitride micron-scale particles (Si_3_N_4_ MPs), cobalt chromium wear particles (CoCr), silicon dioxide nanoparticles (SiO_2_ NPs) and Ti-6Al-4V wear particles.

	Major Diameter (d_max_)	Aspect Ratio	Roundness
Si_3_N_4_ NPs	35.182 ± 0.944 nm	1.301 ± 0.017	0.753 ± 0.009
Si_3_N_4_ MPs	199.366 ± 31.622 nm	1.585 ± 0.066	0.586 ± 0.016
CoCr	21.546 ± 1.321 nm	1.319 ± 0.029	0.677 ± 0.012
SiO_2_ NPs	27.039 ± 1.464 nm	1.248 ± 0.024	0.585 ± 0.018
Ti-6Al-4V	2.829 ± 1.001 μm	1.743 ± 0.131	0.526 ± 0.031

Note: All values expressed as mean ± 95% Confidence Interval.

The micron-scale Si_3_N_4_ particles were mostly submicron in size, while the Ti-6Al-4V alloy particles had a broad size range of 60 nm to 40 µm (Fig. 1). The micron-scale Si_3_N_4_ particles were granular or irregular-angulated, whereas Ti-6Al-4V alloy particles were mostly observed as flakes or shards with a high mean aspect ratio (Fig. 2 and Table 1).

**Figure 3:**
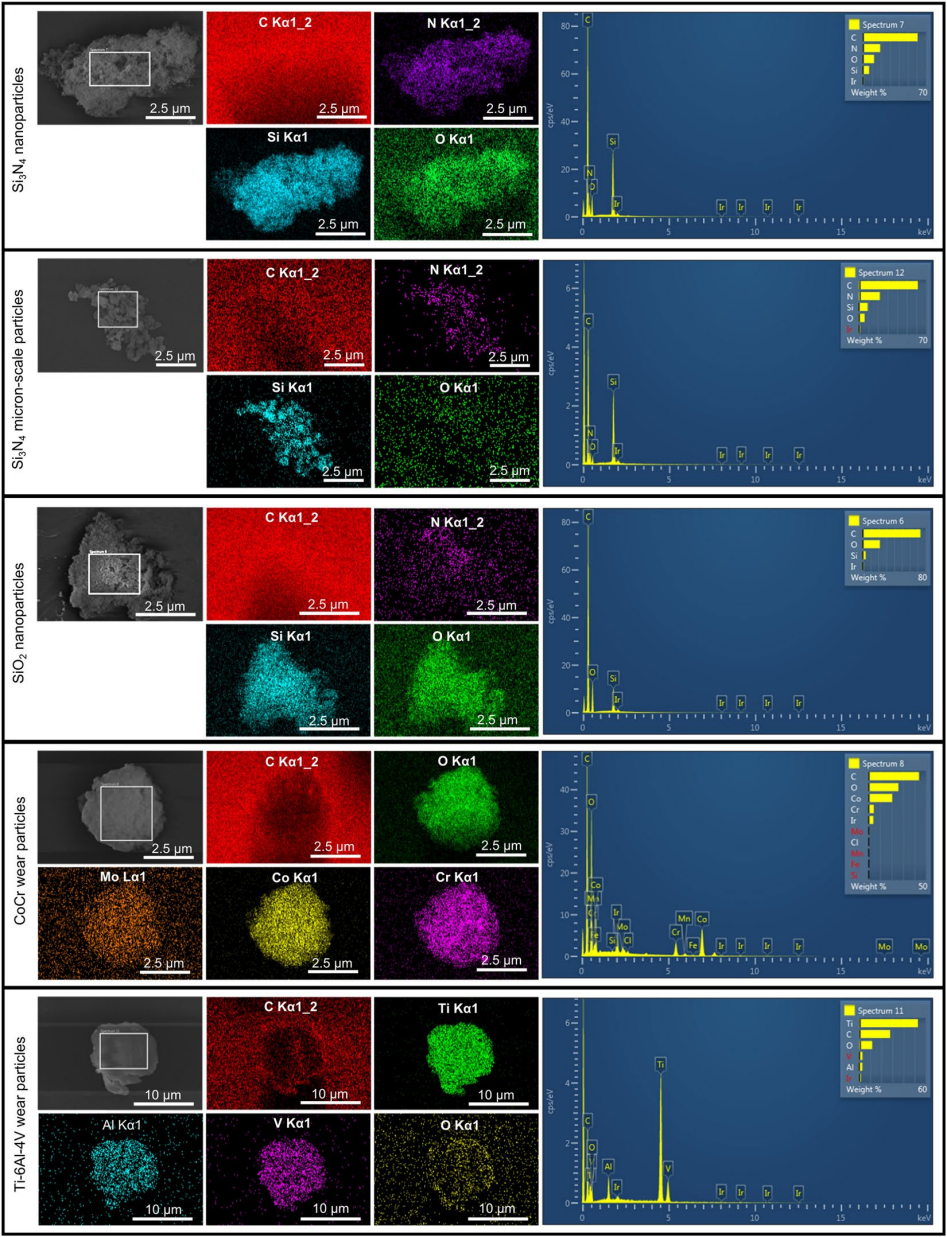
Elemental analysis of Si_3_N_4_ nanoparticles, Si_3_N_4_ micron-scale particles, SiO_2_ nanoparticles, CoCr wear particles and Ti-6Al-4V wear particles. The particles were imaged by scanning electron microscopy (grayscale images) and the corresponding elemental maps (coloured images) were produced by energy dispersive x-ray spectroscopy (EDS). Further, EDS spectra were produced for each particle type from the regions shown as rectangular selections on the grayscale images. Carbon signals originated from the polycarbonate membrane and the iridium signals originated from the coating.

Energy dispersive spectroscopy (EDS) confirmed the presence and composition of the constituent elements for each type of particle (Fig. 3). In addition, both elemental maps and EDS spectra indicated the presence of oxygen in the Si_3_N_4_ nanoparticles, which suggested the presence of oxides on the nanoparticle surface. A minor proportion of oxygen was detected in the EDS spectra of the micron-scale Si_3_N_4_ particles. When compared to the SiO_2_ nanoparticles both type of Si_3_N_4_ particles had lower proportions of oxygen. CoCr particles showed the presence of oxygen in addition to cobalt, chromium, molybdenum and other trace elements, which suggested the presence of chromium oxide (Cr_2_O_3_). Ti-6Al-4V alloy particles showed the presence of titanium, aluminium and vanadium. Low levels of oxygen were also detected indicating potential passivation and formation of an oxide layer.

### The Effects of Si_3_N_4_ and Control Particles on the Viability of PBMNCs

**Figure 4:**
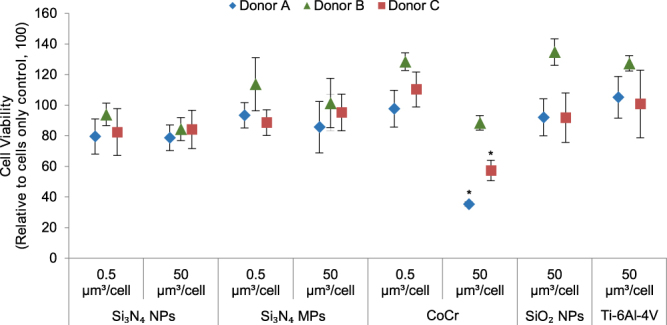
Viability of PBMNCs from donor (**A**), donor (**B**) and donor (**C**) cultured for 24 h with silicon nitride nanoparticles (Si_3_N_4_ NPs), silicon nitride micron-scale particles (Si_3_N_4_ MPs) and cobalt-chromium wear particles (CoCr) at low doses (0.5 μm^3^ particles per cell) and high doses (50 μm^3^ particles per cell); Silicon dioxide nanoparticles (SiO_2_ NPs) and Ti-6Al-4V wear particles (Ti) at high doses (50 μm^3^ particles per cell). Error bars show 95% Confidence Intervals. *Significant difference from the cell only control (ANOVA and Tukey-Kramer post hoc test p < 0.05).

Si_3_N_4_ (both nanoscale and micron-scale), SiO_2_ and Ti-6Al-4V alloy particles cultured with PBMNCs at low doses (0.5 µm^3^ of particles per cell) and high doses (50 µm^3^ of particles per cell) for 24 h had no significant effect on cell viability for all of the donors (Fig. 4). However, CoCr particles at high doses significantly adversely affected the viability of PBMNCs from donor A and donor C (p < 0.05; ANOVA and Tukey-Kramer post hoc test) (Fig. 4). Overall donor-dependent heterogeneity was found to be higher for CoCr particles in comparison to Si_3_N_4_, SiO_2_, or Ti-6Al-4V alloy particles, which is explained further in the Discussion.

### Release of TNF-alpha from Particle-treated PBMNCs

**Figure 5:**
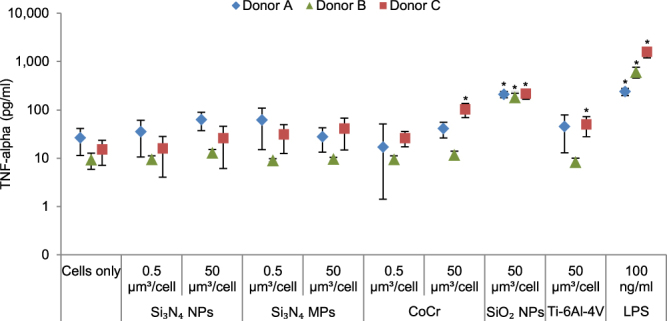
Mean TNF-α release from PBMNCs from donor (**A**), donor (**B**) and donor (**C**) cultured for 24 h with Si_3_N_4_ (nanoscale and micron-scale) and CoCr wear particles at low doses (0.5 μm^3^ particles per cell) and high doses (50 μm^3^ particles per cell); SiO_2_ and Ti-6Al-4V particles at high doses (50 μm^3^ particles per cell). Error bars show 95% Confidence Intervals. *Significant difference from the cell only control (ANOVA and Tukey-Kramer post hoc test, p < 0.05) LPS: Lipopolysaccharide positive control.

When Si_3_N_4_ (both micron-scale and nanoscale) particles were incubated with PBMNCs at low doses (0.5 µm^3^ of particles per cell) and high doses (50 µm^3^ of particles per cell) for 24 h no significant increase in the levels of TNF-α release was observed compared to the cells only control. Conversely, high doses of SiO_2_ particles induced the release of significantly elevated levels of TNF-α after 24 h in PBMNCs from all donors (p < 0.05; ANOVA and Tukey-Kramer post hoc test) (Fig. 5). The positive control (100 ng.ml^−1^ LPS) treated cells also released significantly elevated levels of TNF-α in all the donors, as expected (p < 0.05; ANOVA and Tukey-Kramer post hoc test) (Fig. 5). High doses of CoCr and Ti-6Al-4V wear particles induced the release of significantly elevated levels of TNF-α in PBMNCs from donor C (p < 0.05; ANOVA and Tukey-Kramer post hoc test) (Fig. 5).

### DNA damage in Particle-treated PBMNCs

**Figure 6:**
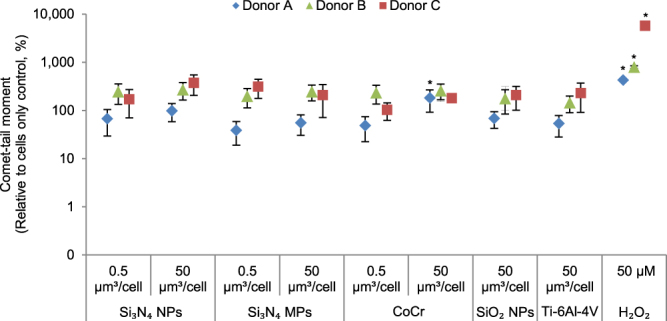
DNA damage in PBMNCs from donor (**A**), donor (**B**) and donor (**C**) cultured for 24 h with Si_3_N_4_ (nanoscale and micron-scale) and CoCr wear particles at low doses (0.5 μm^3^ particles per cell) and high doses (50 μm^3^ particles per cell); SiO_2_ nanoparticles and Ti-6Al-4V alloy particles at high doses (50 μm^3^ particles per cell). H_2_O_2_: Hydrogen Peroxide positive control (50 μM). Error bars show 95% Confidence Intervals. *Significant difference from the cell only control (ANOVA and Tukey-Kramer post hoc test, p < 0.05).

PBMNCs challenged with nanoscale and micron-scale Si_3_N_4_ particles at low and high doses, together with Ti-6Al-4V alloy particles and SiO_2_ nanoparticles both at high doses, did not cause DNA damage above the baseline level (cells only negative control) (Fig. 6). However, CoCr nanoscale wear particles caused significantly elevated comet tail moment compared to the cell only negative control (p < 0.05; ANOVA and Tukey-Kramer post hoc test) (Fig. 6) in PBMNCs from donor A. This indicated significant levels of single- and double-strand breaks in the DNA of PBMNCs from donor A incubated with CoCr wear particles. In addition, the positive control treatment (50 µM H_2_O_2_) caused significantly elevated comet tail moment compared to the cell only negative control (p < 0.05; ANOVA and Tukey-Kramer post hoc test) in all three donors (Fig. 6), as expected.

### Oxidative stress in Particle-treated PBMNCs

**Figure 7:**
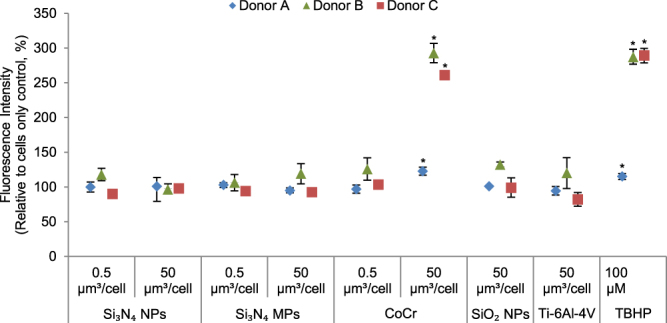
Reactive oxygen species produced in PBMNCs from donor (**A**), donor (**B**) and donor (**C**) cultured for 24 h with Si_3_N_4_ (nanoscale and micron-scale) and CoCr wear particles at low doses (0.5 μm^3^ particles per cell) and high doses (50 μm^3^ particles per cell); SiO_2_ nanoparticles and Ti-6Al-4V alloy particles at high doses (50 μm^3^ particles per cell). TBHP: Tert-butyl Hydroperoxide positive control(100 μM). Error bars show 95% Confidence Intervals. *Significant difference from the cell only control (ANOVA and Tukey-Kramer post hoc test, p < 0.05).

**Figure 8:**
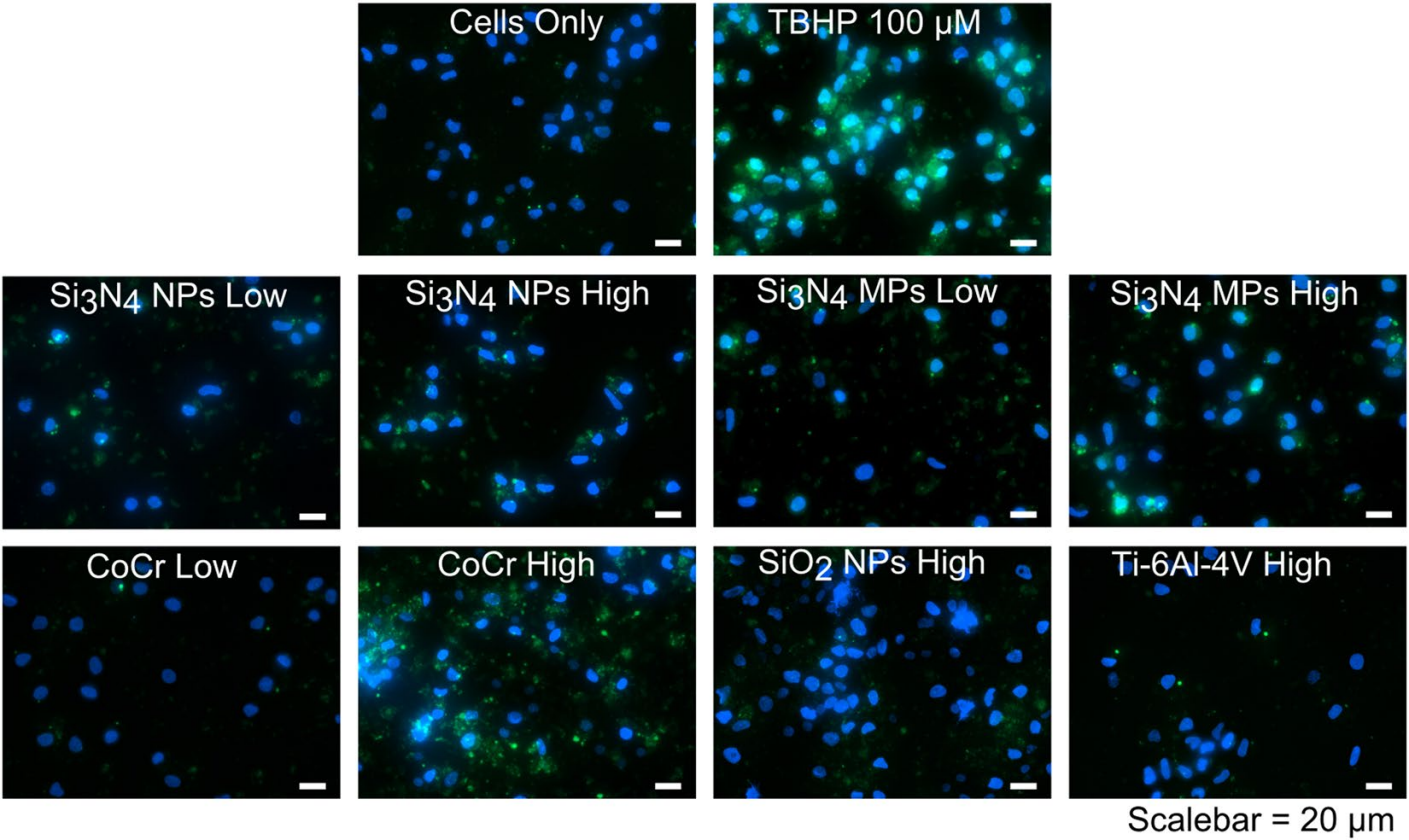
Visualisation of the presence of reactive oxygen species (ROS) in PBMNCs from donor C incubated for 24 h with silicon nitride nanoparticles (Si_3_N_4_ NPs), silicon nitride micron-scale particles (Si_3_N_4_ MPs), CoCr wear particles, silicon dioxide nanoparticles (SiO_2_ NPs) and Ti-6Al-4V alloy particles. Positive control cells were incubated with 100 μM Tert-butyl Hydroperoxide (TBHP). Blue signal represents nuclei (Hoechst dye staining) and green signal represents fluorescent ROS.

No significant reactive oxygen species production was detected from nanoscale or micron-scale Si_3_N_4_ particles, SiO_2_, or Ti-6Al-4V particles; however, once again the CoCr particles caused the release of significantly elevated levels of ROS (p < 0.05; ANOVA and Tukey-Kramer post hoc test) (Figs 7 and 8) in PBMNCs from all of the donors. In addition, the positive control, TBHP, caused significantly elevated levels of ROS production as expected (p < 0.05; ANOVA and Tukey-Kramer post hoc test) (Figs 7 and 8).

## Discussion

New generation orthopaedic biomaterials such as Si_3_N_4_ need to be thoroughly investigated for their potential to induce adverse responses in humans. The present study aimed to evaluate the biological impact of Si_3_N_4_ particles using a novel biological evaluation platform capable of assessing cytotoxicity, osteolytic cytokine release, genotoxicity, and oxidative stress potential simultaneously from a single blood sample of each human donor. PBMNCs from multiple human donors were challenged with a range of test and control particles at clinically relevant low and high doses.

It is widely recognised that the particle size and surface chemistry are key factors influencing the biological interactions of particles with cells. Nanoscale CoCr wear particles generated by modern medical grade alloys are known to disintegrate faster in biological fluids causing short-term adverse effects within the cells, whereas micron-scale CoCr wear particles have a higher propensity of causing a low-level but longer lasting effect within the cells due to prolonged ion release^30,31^. Such differences can only be detected when both nanoscale and micron-scale particles are used for biological evaluation of a slowly dissolving material such as silicon nitride. Moreover, conventional ceramics such as alumina are known to produce both nanoscale and micron-scale wear particles clinically in joint replacements^32^. Therefore, the present study included both nanoscale and micron-scale Si_3_N_4_ particles to evaluate the overall biological impact of Si_3_N_4_ as an orthopaedic biomaterial. Furthermore, SiO_2_ nanoparticles were used in this study for scenarios where it is hypothesized that the Si_3_N_4_ surface might be oxidised to SiO_2_ as shown below in equation 1^9,10^.

1 $$S{i}_{3}{N}_{4}+6{H}_{2}\rightleftharpoons 3Si{O}_{2}+4N{H}_{3}\rightleftharpoons 3Si{O}_{2}+2{N}_{2}+6{H}_{2}$$

Experimentally, this was confirmed by EDX analysis of Si_3_N_4_ and SiO_2_ particles. The presence of oxygen in the EDS spectra of both nanoscale and micron-sized Si_3_N_4_ particles indicated the presence of oxides. Moreover, both elemental mapping and EDX compositional analysis indicated that oxygen was found at higher proportions in SiO_2_ nanoparticles compared to Si_3_N_4_ nanoparticles, which suggested that only outer surfaces of the Si_3_N_4_ nanoparticles were oxidised. Furthermore, Si_3_N_4_ nanoparticles showed higher levels of oxygen in comparison to micron-scale Si_3_N_4_ particles. This confirmed that the larger surface area of the Si_3_N_4_ nanoparticles was responsible for the formation of higher levels of oxides on the particle surface.

CoCr was included in this study as a reference positive particle control due to its known *in vitro* toxicity as wear particles^22,33^. Ti-6Al-4V was included in the study to evaluate the biological impact of metal particles released due to wear at non-articulating interfaces such as taper junctions. Moreover, both CoCr and Ti-6Al-4V are currently being investigated as substrates for ceramic-like silicon nitride coatings^28,29^. Medical grade CoCr and a Ti-6Al-4V alloy were used to generate the CoCr and Ti-6Al-4V wear particles in a pin-on-plate simulator. This method has been shown to produce particles with clinically relevant sizes and morphologies^34,35^.

Presence of the constituent elements in both CoCr and Ti-6Al-4V was confirmed by EDX analysis. Moreover, elemental maps of CoCr particles indicated the presence of oxygen which suggested the presence of Cr_2_O_3_ in the medical grade CoCr alloy, as shown previously^36^.

Use of appropriate cells is important for the clinically relevant evaluation of the biological impact of orthopaedic wear particles. PBMNCs isolated from human donors offer clinically relevant *in-vitro* evaluation of the wear particles, due to their recognised role in the *in-vivo* response to orthopaedic wear particles^37,38^. Therefore, PBMNCs were used in the present study. Moreover, PBMNCs were sourced from three donors for investigating donor-dependent responses.

This study used volumetric concentrations of 0.5 and 50 µm^3^ particles per cell as the low and high particle doses, respectively. A low dose of 0.5 µm^3^ particles per cell was chosen because it is comparable to a low particle load of 2 × 10^−2^ mm^3^/g of tissue (or 1.38 × 10^5^ µg per g of tissue) typically observed in retrievals^39^. A high dose of 50 µm^3^ particles per cell was chosen, as this dose was comparable to the accumulated particle load over the lifespan of a device or due to accelerated wear in a failing device.

**Figure 9:**
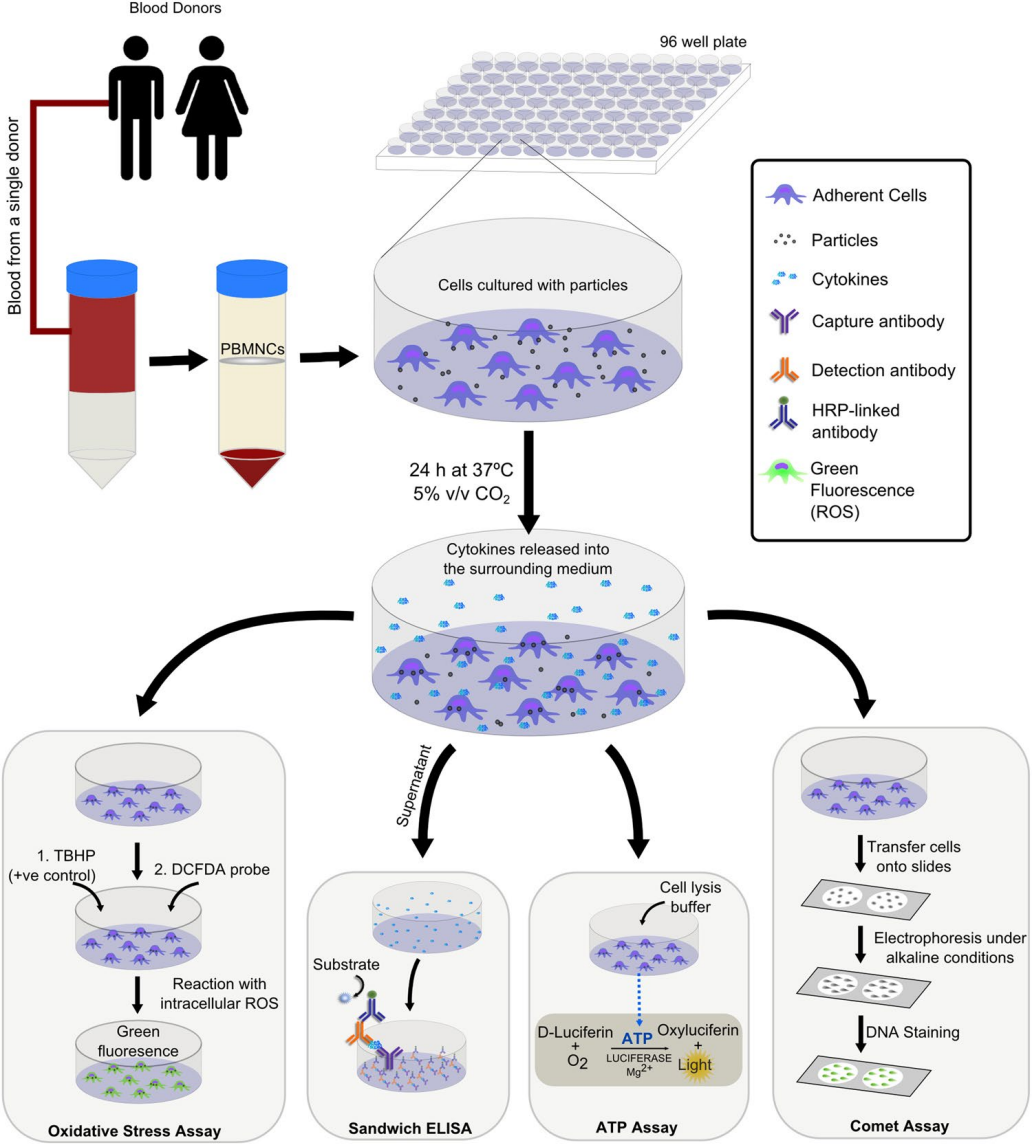
Schematic representation of the biological evaluation platform for testing the biological impact of orthopaedic wear particles in humans. Peripheral blood mononuclear cells isolated from the blood of a single human donor are seeded in 96 well plates and challenged with particles for 24 h at 37 °C in 5% (v/v) CO_2_. Oxidative stress measurements are carried out in black optical bottom 96 well plates using dichlorofluorescein diacetate (DCFDA) as a probe for detecting reactive oxygen species. Osteolytic/inflammatory cytokines are measured using sandwich enzyme-linked immunosorbent assay (ELISA). Cell viability is measured using luciferase based ATP assay. Single and double stranded DNA damage is measured using an alkaline comet assay.

A number of assays were included in this study to elucidate a broad spectrum of cellular responses to Si_3_N_4_ particles. The selection of assays was based on responses previously observed to orthopaedic biomaterials *in vivo*, e.g. CoCr particles have been linked with cytotoxicity, inflammatory cytokine release, genotoxicity, and pseudotumour formation^40–43^ whereas UHMWPE wear particles have been mainly associated with chronic cytokine release leading to osteolysis. TNF-α is one of the key cytokines associated with the inflammatory response^44^. Therefore, in addition to ATPlite assay for cytotoxicity testing, TNF-α cytokine release determined by ELISA and genotoxicity measured by comet assay were included. Furthermore, oxidative stress was included as a unifying factor to determine the toxicity and carcinogenicity potential of a material. Excess generation of reactive oxygen species has been associated with cytotoxicity, DNA damage, cell membrane damage by lipid peroxidation, the release of inflammatory cytokines, and unwanted extracellular matrix deposition^45^. A sensitive and efficient biological evaluation platform was developed to perform these assays using the PBMNCs isolated from a single blood sample from each human donor (Fig. 9). More specifically, a high-throughput format was developed for each assay in the form of 96-well cell culture plates for all the assays to conserve the number of cells per treatment, coupled with the use of high-throughput slides for the comet assay and automatic measurement and quantification of the release of ROS using a fluorescence plate reader.

The ATPlite assay was used for cytotoxicity testing of the particles due to its high sensitivity in detecting ATP as an indicator of the cell viability. Si_3_N_4_ particles cultured with PBMNCs did not affect the cell viability at either low doses (0.5 μm^3^ particles per cell) or high doses (50 μm^3^ particles per cell) for any of the donors. This is in agreement with previous studies where the viability of murine L929 fibroblasts^2^, human MG-63 osteoblast-like cells^3,25^, and human fibroblasts^26^ were not reduced in the presence of Si_3_N_4_. CoCr wear particles significantly reduced the viability of PBMNCs from donor A and donor C, indicating a donor-dependent response. The cytotoxic response to CoCr wear particles was in agreement with the previous studies^22^. Moreover, genetic variability within the genes encoding inflammation and hypoxia-related biochemical mediators could be the reason behind the variable cytotoxic responses to CoCr wear debris observed in this study^46,47^. Extensive but variable necrosis has also been reported as a distinguishing feature of adverse reactions to metal debris (ARMD) in tissues from failed metal on metal implants^48^.

Further evaluation of the biological responses to Si_3_N_4_ particles by measuring TNF-α proinflammatory cytokine release by ELISA did not show any significant release of TNF-α. A previous study by Zhang *et al*. observed significantly increased levels of TNF-α released by osteoblast-like MG63 cells in the presence of Si_3_N_4_ nanoparticles^25^. However, this difference in the responses may be due to the different cell types used in the two studies.

PBMNCs from all the donors released significantly higher levels of TNF-α when incubated with high doses of SiO_2_ nanoparticles. Since the size and morphology of both SiO_2_ and Si_3_N_4_ nanoparticles were similar, the observed differences in the levels of TNF-α was believed to be indicative of differences in their surface compositions. Production of inflammatory cytokines by PBMNCs in the presence of SiO_2_ nanoparticles has been observed previously^49^. Moreover, recent studies have postulated that the dominant chemical species present on the particle surface or released into the surrounding fluids is orthosilicic acid as shown below^9,50^.

2 $$3Si{O}_{2}+6{H}_{2}O\rightleftharpoons 3Si{(OH)}_{4}$$

Moreover $N{H}_{3}$ released by the oxidation of silicon nitride particles (Eq. 1) could react with ${H}_{2}O$ to form ${{{NH}}_{4}}^{+}$ ions as shown below^8^.

3 $${H}_{2}O+N{H}_{3}\rightleftharpoons O{H}^{-}+N{{H}_{4}}^{+}$$

Release of statistically significant levels TNF-α was also observed for CoCr and Ti-6Al-4V wear debris. However, this response was only observed in one out of three donors for both type of particles, which indicates that inflammatory responses in PBMNCs are donor dependent.

The comet assay was used for evaluating DNA damage in PBMNCs exposed to particles. Si_3_N_4_ particles (nanoscale and micron-scale) did not cause any damage to the DNA at either low or high doses, whereas PBMNCs from donor A showed significant damage in both single and double-stranded DNA in the presence of high doses of nanoscale CoCr wear particles, which is in agreement with previous *in vitro* and *in-vivo* studies^30,31,51,52^. Moreover, donor A also had the highest cytotoxic response to CoCr particles (over 50% reduction in the cell viability) (Fig. 4). As observed in a previous study, this might suggest that orthopaedic metals usually only cause DNA damage when they are highly cytotoxic^53^.

Oxidative stress in PBMNCs was measured using 2′,7′-dichlorodihydro-fluorescein diacetate (DCFDA) as a probe for measuring intracellular ROS. No significant increase in the production of ROS in PBMNCs was observed for Si_3_N_4_ particles, SiO_2_ nanoparticles and Ti-6Al-4V wear particles after 24 h. However, CoCr and the positive control (Tert -Butyl hydroperoxide) significantly increased the production of ROS after 24 h, as observed in a previous study^30^. Moreover, oxidative stress in PBMNCs induced by CoCr particles could be attributed to diverse mitochondrial ROS-dependent responses^30^.

Biological responses of PBMNCs to particles used in this study showed varying degrees of donor-dependent heterogeneity. Although PBMNCs are predisposed to inter-donor variability^54^, the present study observed greater donor variation in the cytotoxicity assay and release of ROS induced by CoCr particles. Additionally, CoCr particles caused DNA damage and significant levels of TNF-α release in only one out of three donors. Such donor-dependant variations are potentially associated with the genetic variability within the genes encoding a range of biochemical mediators involved in particle-induced cytotoxicity, inflammatory response, oxidative stress and genotoxicity in the immune cells. The most common type of such genetic variations is single nucleotide polymorphisms (SNPs). A previous study has reported an association between the SNP at the –238 position in the TNF gene promoter region of the TNF gene and osteolysis after cemented total hip arthroplasty (THA) in 481 subjects^55^. Another study has found the protective effect of carriage of IL1RA +2018C allele against osteolysis after cemented THA and a positive association of this allele with IL-1Ra mRNA production in PBMNCs challenged with titanium particles^56^. Clinically, genetic variations potentially lead to heterogeneity in adverse responses such osteolysis in metal-on-polyethylene implants^55^, and may also cause variability in necrosis, inflammatory cell infiltration, or aseptic lymphocytic vasculitis associated lesions (ALVAL) in patients with similar levels of metal-on-metal implant wear^42,43^.

One limitation of the present study was the short duration of the culture of PBMNCs with particles (up to 24 h), which cannot predict the long-term effect of particles. However, 24 h time point was chosen due to the limited lifespan of PBMNCs in the current *in vitro* cell culture conditions. Another limitation of this study was the use of commercially available Si_3_N_4_ particles. However, the particles were selected based on their similar physicochemical characteristics to those observed in the preliminary wear analysis of the Si_3_N_4_ bearings^15^. Donor variability was also considered as a limitation for determining conclusive responses of PBMNCs to the test and control particles.

This study demonstrates that the biological impact of orthopaedic wear particles can be evaluated using PBMNCs isolated from multiple donors, challenged with a careful selection of test and control particles, and tested using assays that are linked to the responses observed *in vivo*. Detailed conclusions are summarised as follows:

1. A novel biological evaluation platform was introduced for testing cytotoxicity, inflammatory cytokine release, oxidative stress and DNA damage from a single blood sample obtained from each donor.
2. The biocompatibility of Si_3_N_4_ particles was demonstrated at clinically relevant low and high volumetric concentrations of particles per cell, for both nanoscale and micron-scale particles.
3. Significant differences in the levels TNF-α released by the PBMNCs treated with silicon dioxide nanoparticles and Si_3_N_4_ nanoparticles suggest that the surface composition is different for both types of particles.
4. CoCr wear particles induced donor dependent toxicity in PBMNCs at high volumetric concentrations of particles. This included reduction in cell viability, significant release of TNF-α, significant increase in the oxidative stress, and significant damage to the single and double-stranded DNA.
5. Ti-6Al-4V wear debris had lower biological impact than CoCr. However, depending on the donor, there could be a significant release of TNF-α at high volumetric concentrations of particles.

## Methods

### Preparation of endotoxin-free Si_3_N_4_ and SiO_2_ model particles

Commercially available Si_3_N_4_ particles (<50 nm and <1 µm, Sigma UK) and SiO_2_ nanoparticles (<100 nm, Sigma UK) were heat-treated for 4 h at 180 °C to destroy endotoxins and sterilise the particles.

### Preparation of endotoxin-free CoCr and Ti-6Al-4V wear particles

CoCr pins and plates were manufactured from medical grade cobalt-chromium alloy (ASTM F1537) with a low carbon content (<0.2% wt) and their contact surfaces were polished to a smooth finish (R_a_ 0.01–0.02 µm). Ti-6Al-4V pins and plates were manufactured from medical grade Ti-6Al-4V alloy (ASTM F1108) and their contact surfaces were polished to a smooth finish (R_a_ 0.01–0.02 µm), as described previously^22^.

Wear particles were generated in sterile water (Baxter, UK) in a six-station multidirectional pin-on-plate tribometer, as described previously^22^. Ti-6Al-4V particle suspensions were collected after 60,000 cycles and CoCr particle suspensions were collected after 330,000 cycles. The particle suspensions were stored at −20 °C, following which particle characterisation and cell culture experiments were performed.

For cell culture experiments, CoCr and Ti-6Al-4V particle suspensions were thawed at 37 °C and heat-treated for 4 h at 180 °C to evaporate the water, destroy endotoxins and sterilise the particles.

### Characterisation of the particles by Scanning Electron Microscopy

Particles were re-suspended in sterile water to produce 1 mg.ml^−1^ particle suspensions for each particle type. Dispersion of the particles was carried out by stirring and sonicating the particle suspensions simultaneously for 10 min in an ultrasonic bath (80 watts; 45 kHz ultrasonic frequency; VWR, UK). A 20 µl aliquot was further diluted to 0.002 mg.ml^−1^ with sterile water (final volume 10 ml) and dispersed by using the method mentioned above for the 1 mg.ml^−1^ particle suspensions. Particles were then collected on 0.015 μm pore size polycarbonate membrane filters using a vacuum filtration unit. Filters were dried using an infra-red lamp for 2 h and imaged using a Hitachi SU8230 cold-field-emission scanning electron microscope (CFE-SEM) at 100,000x magnification for nanoscale particles, and at 1000x to 10,000x magnifications for micron-scale particles. An Aztec Energy-Dispersive X-ray system, with a high-resolution detector (80 mm^2^ X-Max SDD, Oxford) was used in combination with the CFE-SEM imaging for elemental analysis of the particle samples. Particle images were analysed using ImageJ (Version 1.44) and mean feret diameter, aspect ratio, and roundness were calculated and expressed as mean ± 95% confidence interval for each material as shown in the Table 1.

### Isolation of PBMNCs from whole blood

Peripheral blood was collected by venepuncture into heparin-coated tubes from three healthy human donors (Faculty of Biological Sciences ethical committee approval BIOSCI 10–108, University of Leeds). Prior to collection of the blood samples, informed consent was obtained from all donors. Donor anonymity was maintained in accordance with the Human Tissue Act 2004. The three donors were labelled as Donor A, Donor B and Donor C. Age, gender, and PBMNC count of these donors are shown in Table 2. PBMNCs were isolated (within 2 h of collecting blood) using Lymphoprep^®^ as a density gradient medium, as described previously^24^.

**Table 2:** Age, gender and PBMNC count of the three human blood donors.

Donor	Gender	Age	PBMNCs/ml
Donor A	F	20–30	1.02 × 10^6^
Donor B	M	20–30	1.39 × 10^6^
Donor C	F	20–30	1.00 × 10^6^

### Preparation of particles for culture with cells

Sterile particles were re-suspended in sterile water (Baxter, UK) under aseptic conditions to make 1 mg.ml^−1^ particle suspensions for each material. Ultrasonic agitation for 10 min was used to disperse the particles, followed by immediate addition of the particle suspensions at various doses to RPMI culture media (Gibco, UK) supplemented with 10% (v/v) foetal bovine serum (Gibco, UK), 2 mM L-glutamine (Sigma, UK), 100 µg.ml^−1^ streptomycin (Sigma, UK), and 100 U.ml^−1^ penicillin (Sigma, UK).

### Culture of PBMNCs with particles

PBMNCs isolated from a single blood sample drawn from each donor were used to setup cytotoxicity, TNF-α release, DNA damage and intracellular reactive oxygen species assays (Fig. 8). Complete RPMI 1640 cell culture media was prepared by supplementing RPMI 1640 media with 10% (v/v) foetal bovine serum, 2 mM L-glutamine, 100 µg.ml^−1^ streptomycin, and 100 U.ml^−1^ penicillin. The PBMNCs were seeded at 1 × 10^5^ cells per ml into three separate cell culture plates: one clear flat-bottom 96-well cell culture plate (Nunc, UK) for the cell viability assay/cytokine release assay; one clear flat-bottom 96-well cell culture plate for the DNA damage assay; and one Black 96-well optical-bottom culture plate (Nunc, UK) for the oxidative stress assay. The RPMI 1640 cell culture media was added to the cells and the culture plates were incubated for 12 h in 5% (v/v) CO_2_ at 37 °C to allow the attachment of mononuclear phagocytes to the bottom surface of each well. Non-adherent cells were removed by washing the cells with warm PBS (Gibco, UK). Si_3_N_4_ and CoCr particles dispersed in complete RPMI 1640 culture media were then added to the adherent phagocytes at a low volume concentration of 0.5 µm^3^ particles per cell and a high volume concentration of 50 µm^3^ particles per cell. Other additional control particles (Ti-6Al-4V and SiO_2_) were added at a high volume concentration of 50 µm^3^ particles per cell. Cells alone were used as a negative control for all the assays. Lipopolysaccharide (LPS; 100 ng/ml) was added to the positive control wells of the cytokine release assay cell culture plate to induce the release of TNF-α. All cells were cultured for 24 h in complete RPMI 1640 culture medium in 5% (v/v) CO_2_ at 37 °C. Positive control wells for the comet and oxidative stress assay culture plates were treated with DNA damage and oxidative stress inducing compounds, respectively, as described in the subsequent sections for these assays.

### Cell viability assay

The viability of the cells incubated with particles was measured using a luminescence-based ATPlite assay (Perkin Elmer, UK). Cells were lysed and treated with the substrate (provided in the ATPlite kit). The contents of 96-well cell culture plates were separately transferred to 96-well white plates. A 96-well plate reader (Plate Chameleon, Hidex Finland) was used to measure and quantify the luminescence for each well after dark-adapting the plates for 10 minutes. Data were expressed as mean counts per second (cps) ±95% confidence intervals (n = 4).

### TNF-α release by ELISA

Culture supernatants were collected after 24 h and stored at −80 °C and TNF-α release was measured by sandwich ELISA following the manufacturer’s instructions (Diaclone, UK). Results were expressed as mean TNF-α concentration (pg.ml^−1^) ±95% confidence intervals (n = 4).

### Measurement of DNA damage using the comet assay

Single and double-stranded DNA damage in response to incubation with particles was measured in PBMNCs after 24 h using the alkaline comet assay (Tevigen, UK). A high throughput approach was developed to analyse 20 samples in a single experiment using HT20 comet slides (Tevigen, UK). Positive control wells were incubated for 5 min with hydrogen peroxide (50 µM) to induce DNA damage to the cells. The adherent PBMNCs were detached from the 96-well cell culture plates by using a rubber policeman (UltraCruz narrow blade cell lifter, Santa Cruz Biotechnology USA). Cells were then washed with ice-cold PBS and collected by centrifugation at 200 g for 10 min at 4 °C.

Cells exposed to each particle material were mixed with 1% (w/v) Low-Melting-Point agarose (Invitrogen). Agarose gels were prepared in duplicate for each treatment on the HT20 slides (Tevigen, UK). A series of steps consisting of cell lysis, unwinding of DNA, followed by single cell gel electrophoresis were performed as recommended by the comet assay manufacturer. SYBR Gold was used to stain DNA and comets were imaged using a high throughput tiled imaging system using an optical microscope (Zeiss, UK). Images were analysed using Comet IV software (Perceptive Instruments, UK) and the tail moment was measured for each comet. Data were expressed as mean comet-tail moment ±95% confidence intervals (n = 4).

### Particle-induced oxidative stress measurement

Intracellular reactive oxygen species (ROS) generation was measured in PBMNCs 24 h after exposure to various particles using the ABCAM Cellular ROS detection kit (Abcam, UK). 2′,7′ -dichlorofluorescein diacetate (DCFDA) was added to each well at a working concentration of 25 μM and the cell culture plates were incubated for 45 min at 37 °C for the diffusion of DCFDA into the cells. Cells only were used as a negative control and positive control wells were treated with 100 µM Tert-butyl Hydroperoxide (TBHP) for 2 h at 37 °C to induce the production of ROS. Intracellular DCFDA was then deacetylated by cellular esterases and oxidized by ROS into fluorescent 2′,7′ –dichlorofluorescein. The fluorescence measurements were taken using a 96 well plate reader (Plate Chameleon, Hidex Finland). Images were also captured to visualise the generation of ROS in the cells using a fluorescence microscope (Zeiss, UK). Data were expressed as mean fluorescence ± 95% confidence intervals (n = 4).

### Statistics

Particle size and shape descriptors were expressed as mean ± 95% confidence intervals. Cell viability, cytokine release, oxidative stress, and comet assay data were expressed as mean ± 95% confidence intervals and the data was analysed using one-way analysis of variance (ANOVA, p < 0.05) and Tukey-Kramer post-hoc analysis at a significance level of 0.05 (SPSS Statistics Version 22, IBM Corp. USA).

The datasets generated during the current study are available from the corresponding author on reasonable request.
